# Tinnitus and risk of Alzheimer’s and Parkinson’s disease: a retrospective nationwide population-based cohort study

**DOI:** 10.1038/s41598-020-69243-0

**Published:** 2020-07-22

**Authors:** Hsuan-Te Chu, Chih‐Sung Liang, Ta-Chuan Yeh, Li-Yu Hu, Albert C. Yang, Shih-Jen Tsai, Cheng-Che Shen

**Affiliations:** 1 Department of Psychiatry, Beitou Branch, Tri-Service General Hospital, National Defense Medical Center, Taipei, Taiwan; 2 Institute of Brain Science, National Yang-Ming University, Taipei, Taiwan; 3 Department of Psychiatry, Tri-Service General Hospital, National Defense Medical Center, Taipei, Taiwan; 4 Graduate Institute of Medical Sciences, National Defense Medical Center, Taipei, Taiwan; 5 Department of Psychiatry, Taipei Veterans General Hospital, No 201, Sec 2. Shi-Pai Rd., Taipei, 11217 Taiwan; 6 School of Medicine, National Yang-Ming University, Taipei, Taiwan; 7 Division of Interdisciplinary Medicine and Biotechnology, Beth Israel Deaconess Medical Center/Harvard Medical School, Boston, MA USA; 8 Department of Psychiatry, Chiayi Branch, Taichung Veterans General Hospital, Chiayi, Taiwan

**Keywords:** Neurological disorders, Psychiatric disorders, Risk factors

## Abstract

Tinnitus has been implied as a “soft” sign of neurodegenerative disease, which is characterized by progressive loss of neuronal function, such as Alzheimer’s disease (AD) and Parkinson’s disease (PD). This study aimed to determine whether the risk of developing AD/PD increases after having tinnitus. We conducted a retrospective matched cohort study with 12,657 tinnitus patients and 25,314 controls from the National Health Insurance Research Database (NHIRD) in Taiwan with almost 10 years follow-up. Tinnitus-related risk on developing AD/PD followingly was determined by the Cox regression to identify potential confounding factors. Through the 10-year follow-up period, 398 individuals with tinnitus (3.1%) and 501 control individuals (2.0%) developed AD (*P* < 0.001), and 211 tinnitus patients (1.7%) and 249 control patients (1.0%) developed PD (*P* < 0.001). Compared with controls, patients with tinnitus were 1.54 times more likely to develop AD (95% confidence interval (CI) 1.34–1.78, *P* < 0.001) and 1.56 times more likely to develop PD (95% CI 1.29–1.89, *P* < 0.001), after adjusting confounding factors. Our results indicate an association between tinnitus and higher risk of developing AD and PD. Additional physical comorbidities may also increase the risk of developing AD and PD.

## Introduction

Tinnitus is the sensation of sound without any external acoustic sound source (phantom perception of sound) which is related to altered auditory perception pathway^1^. According to a large-scale study, the overall prevalence of tinnitus in the adult population was reported to be around 10%^2^. The prevalence of tinnitus was higher (12–18%) in people over 60 years of age^3^. Chronic tinnitus usually coexist with a hearing impairment^4^. Various brain functions, such as learning, emotion, memory, concentration, and behavior have been linked to the neural correlates of sensory processing for tinnitus^5^. For a specific group of individuals diagnosed with a central-type tinnitus, evidence refers tinnitus as a “soft” sign of neurodegenerative central nervous system disease^6^.

Parkinson’s disease (PD) and Alzheimer’s disease (AD) are neurodegenerative diseases, which are characterized by gradual progressive loss of neuronal function in an anatomical or physiological way. Up to the present moment, aging is the highest known risk factor for neurodegenerative diseases. Oxidative stress and mitochondrial dysfunction are found in the early stages of total major neurodegenerative diseases^7^. The most common sensory disorder in the elderly is the age-related hearing disability^8^, where mitochondrial dysfunction as well as oxidative stress may play a role in the underlying pathology of the disorder^9^. Evidence indicated that the neuroanatomical changes in the brains of semantic dementia patients may implicate cortico-subcortical auditory and limbic network in the pathogenesis of abnormal auditory percepts^10^.

Previous studies have shown that central auditory dysfunction without severe peripheral hearing loss was associated with higher incidences of cognitive decline and Alzheimer’s dementia^11,12^. Both hearing loss and central auditory dysfunction are associated with higher risk of developing dementia 5–10 years later^13^. Uhlmann et al.^14^ demonstrated an association of hearing impairment and Alzheimer’s dementia. There may be a pathophysiological interplay of hearing impairment and neurodegeneration. Although the prevalence of tinnitus is not exactly related to the prevalence of hearing loss, chronic tinnitus is usually accompanied by a hearing impairment^4^. Previous research revealed that chronic tinnitus was related to the anatomical brain abnormalities, including cortical grey matter reduction and reduced white matter integrity^15^. In previous decades, structural and functional brain research showed the correlation between annoying tinnitus and various forms of cognitive dysfunction, especially executive control of attention and working memory^16^. Such studies suggest that changes in hearing, olfaction may antecede the beginning of cognitive impairments and dementia as the strong risk factors of AD dementia^17,18^. On the other hand, adequate research shows that specific sensory changes may be early biomarkers for AD^13^. Previous studies have not used population-based cohort study to evaluate the relationship between tinnitus and subsequence risk of AD and PD.

We applied nationwide data from the National Health Insurance (NHI) system to operate a retrospective population-based cohort study in Taiwan. This reliable data has been used for various studies, including tinnitus and AD/PD^19–21^. The hypothesis of this study is that tinnitus might be related with the likelihood of subsequent diagnosis of AD and PD. We aim to determine the possibility of an association between tinnitus and a higher risk of AD and PD development.

## Methods

### Data sources

The NHI program, an essential health insurance program instituted in 1995, provided comprehensive medical care coverage to about 99% of the residents in Taiwan, including inpatient, outpatient, emergency, and traditional Chinese medicine services^22^. Comprehensive information on medical visits, including diagnostic codes and prescription details according to the International Classification of Diseases, Ninth Revision, Clinical Modification (ICD-9-CM) are provided by the NHI Research Database (NHIRD). NHIRD is supervised by the National Health Research Institutes, with confidentiality kept in congruence with the directives of the NHI Bureau, Taiwan. Data used in the current study are from the Longitudinal Health Insurance Database 2005 (LHID2005). LHID2005 data were methodically and randomly retracted from the NHIRD, which contains data on one million people. No significant differences in the average insured payroll-related amount, sex distribution, or age distribution between patients in the LHID2005 and those in the original NHIRD were reported by NHRI^23^.

### Ethics statement

Approval for the present study was obtained from the Institutional Review Board of the Taipei Veterans General Hospital, and all methods were carried out in accordance with relevant guidelines and regulations. As the NHI data set is comprised of de-identified secondary data for research directions, written consents from study participants was unnecessary. A formal written waiver for the demand for consent was issued by the Institutional Review Board of Taipei Veterans General Hospital.

### Study population

A retrospective cohort study was carried out using the LHID2005 data, which is made up of patients (20 years and older) lately diagnosed with tinnitus between January 1st, 2000 and December 31st, 2004. Definition of tinnitus was based on ICD-9-CM Code 388.3 in Ambulatory care expenditures by visits (CD) and Inpatient expenditures by admissions (DD) files of LHID 2005. To ensure diagnostic validity and patient homogeneity, we collected only patients diagnosed by otolaryngologists and neurologists, with at least two consistent tinnitus diagnoses to improve diagnostic validity. Similar methods for the identification of AD and PD had been applied in previous studies^24,25^. In Taiwan, PD is usually diagnosed by neurologists and two of the four main symptoms (shaking or tremor; slowness of movement, called bradykinesia; stiffness or rigidity of the arms, legs or trunk; trouble with balance and possible falls, also called postural instability) must be present over a period of time to consider a PD diagnosis. According to previous studies, the PD diagnosis included in the NHI claims is considered valid (the sensitivity, specificity, positive predictive value and negative predictive value were 97.6%, 92.3%, 98.8% and 85.7%, respectively)^26,27^. Similarly, AD is usually diagnosed by neurologists or psychiatrists in Taiwan. A worsening of cognitive functions, such as memory, and executive function etc., from a preexisting individual level is the main symptom of AD and the impairment of cognitive function is severe enough to impair a person's ability to perform everyday activities. Some rating scales, such as Mini-Mental State Examination and Clinical Dementia Rating, will be performed to evaluate the patient’s cognitive and daily function. Serial blood tests and brain image will be arranged to rule out other possible causes of cognitive impairment. Prior to enrollment, we ruled out patients diagnosed with Parkinson disease (ICD-9-CM Code 332) or Alzheimer disease (ICD-9-CM Codes 290.0, 290.3, and 294.1). We randomly selected 2 sex- and age-matched control patients from the LHID 2005 without tinnitus, PD, or AD diagnoses for every tinnitus patient involved in the final cohort. We observed all tinnitus and control patients until (1) diagnosed with Alzheimer disease by a psychiatrist or a neurologist, (2) diagnoses with Parkinson disease by a neurologist, (3) death, or (4) December 31, 2009. The primary clinical outcome evaluated was psychiatrist- or neurologist- diagnosed Alzheimer disease and neurologist- diagnosed Parkinson disease.

### Statistical analyses

We calculated the incidence of newly diagnosed Parkinson disease and Alzheimer disease in the patient groups. Demographic features including age, sex, typical comorbidities, urbanization, and monthly income, were compared between the tinnitus and control groups in our study using chi-squared and independent *t* tests. Personal characteristics, including age, sex, monthly income, and urbanization, were extracted from Registry for beneficiaries (ID) files of LHID 2005. Calculations based on the total income of beneficiaries, the monthly income of patients based on their insurance premium was supposed. Monthly income was grouped into low (monthly income < NT$20,000 per month), median (> NT$20,000 < NT$40,000 per month), and high income (≥ NT$40,000 per month) levels. Urbanization was subdivided into three groups: urban, suburban, and rural. Urbanization and monthly income levels were used to imply socioeconomic status. Typical comorbidities, comprising hypertension (ICD-9-CM Codes 362.11, 401–405, 437.2), diabetes mellitus (ICD-9-CM Codes 250, 357.2, 362.0, 366.41), coronary artery disease (ICD-9-CM Codes 410–414), congestive heart failure (ICD-9-CM Codes 428.0, 428.43, 428.9, and 398.91), chronic lung disease (ICD-9-CM Codes 490–496, 500–505, and 506.4), head injury (ICD-9-CM Codes 800–804, and 850–854), cerebrovascular disease (ICD-9-CM Codes 430–438), malignancy (ICD-9-CM Codes 140–208, 230–231, and 233–234), osteoarthritis (ICD-9-CM Code 715), and rheumatologic disease (ICD-9-CM Codes 725, 714.0, 714.1, 714.2, 710.0, 710.1, 710.4, and 714.81) were extracted from CD and DD files of LHID 2005 based on ICD-9-CM Codes. Comparison of variables predicting Parkinson disease or Alzheimer disease in both patient groups was made using a stratified cox proportional-hazards regression method which incorporated a robust sandwich-type variance estimator and the stratified cox proportional-hazards regression method was stratified on each matched-pair. The stratified cox proportional-hazards regression method is a modification of the Cox proportional hazards model that allows for control by stratification of a predictor that does not satisfy the proportional hazards assumption. The sandwich-type variance estimator, often known as the robust covariance matrix estimator or the empirical covariance matrix estimator, has achieved increasing use with the growing popularity of generalized estimating equations. Its virtue is that it provides consistent estimates of the covariance matrix for parameter estimates even when a parametric model fails to hold, or is not even specified^28^. For sensitive analyses, we defined 3 models which included different variables in the stratified cox proportional-hazards regression method. In model I, only tinnitus was included in the cox proportional-hazards regression method. In model II, all variables, including tinnitus, typical comorbidities, urbanization, and monthly income, were included as variates in the univariate model. Then, we included univariate analysis factors with a moderate statistically significant relationship (i.e., *P* <  0.1) into a multivariate Cox proportional-hazards regression model by using the forward selection method^29^. In model III, all variables were included into a multivariate Cox proportional-hazards regression directly. Furthermore, we also tested the proportionality of hazards in our work by using Scaled Schoenfeld Residuals test.

We used the Perl programming language (Version 5.12.2) to extract and analyze the data. Data linkage, processing, as well as control sampling were carried out by employing Microsoft SQL Server 2005 (Microsoft Corp., Redmond, WA, USA). All statistical analyses were done using SAS (Version 9.2; SAS Institute Inc., Cary, NC, USA) and SPSS (Version 19.0 for Windows; IBM Corp., New York, NY, USA). Relationships were considered statistically significant when *P* < 0.0.

### Ethics approval and consent to participate

The present study was approved by the Institutional Review Board of the Taipei Veterans General Hospital (2018-07-016AC). Obtaining written consent from the study participants was unnecessary because the NHI data set comprises de-identified secondary data for research directions. The Institutional Review Board of Taipei Veterans General Hospital issued a formal written waiver for the demand for consent.

## Results

### Participant selection

Out of the sample of 12,657 tinnitus patients and 25,314 control patients without tinnitus, 52.4% were women. The median age at recruitment was 52 years (interquartile range [IQR] 41–64 years), and the median follow-up periods for the tinnitus and control patients were 7.42 (IQR 6.09–8.71 years) and 7.40 years (IQR 6.07–8.70 years), respectively. There is a higher frequency of reporting in the tinnitus patients than controls of comorbidities including diabetes mellitus, congestive heart failure, hypertension, head injury, malignancy, cerebrovascular disease, chronic lung disease, osteoarthritis, and rheumatologic disease. During the study period, 398 tinnitus patients (3.1%) and 501 control patients (2.0%) were diagnosed with Alzheimer’s disease (*P* <  0.001). On the other hand, 211 tinnitus patients (1.7%) and 249 control patients (1.0%) were diagnosed with Parkinson’s disease (*P* <  0.001). Demographic and clinical variables of the tinnitus and control patients are presented in Table 1.

**Table 1:** Baseline characteristics of patients with and without tinnitus.

Demographic data	Patients with tinnitus *n* = 12,657		Patients without tinnitus *n* = 25,314		*P* value
	*n*	%	*N*	%	
**Age (years)**^**a**^	52 (41–64)		52 (41–64)		0.982
≥ 60	8,352	66.0	16,704	66.0	
< 60	4,305	34.0	8,610	34.0	
**Sex**					0.999
Male	6,028	47.6	12,056	47.6	
Female	6,629	52.4	13,258	52.4	
**Comorbidities**					
Hypertension	4,047	32.0	6,530	25.8	< 0.001*
Diabetes mellitus	2,219	17.5	3,453	13.6	< 0.001*
Coronary artery disease	179	1.4	299	1.2	0.055
Congestive heart failure	523	4.1	756	3.0	< 0.001*
Chronic lung disease	2,389	18.9	3,492	13.8	< 0.001*
Malignant neoplasms	283	2.2	395	1.6	< 0.001*
Head injury	2030	16.0	3,158	12.5	< 0.001*
Cerebrovascular disease	983	7.8	1,357	5.4	< 0.001*
Osteoarthritis	3,448	27.2	4,799	19.0	< 0.001*
Rheumatologic disease	1,134	9.0	1,422	5.6	< .001*
**Degree of urbanization**					0.227
Urban	7,374	58.3	14,940	59.0	
Suburban	3,974	31.4	7,697	30.4	
Rural	1,309	10.3	2,677	10.6	
**Income group**					< 0.001*
Low income	6,089	48.1	13,002	51.4	
Medium income	4,898	38.7	9,343	36.9	
High income	1,670	13.2	2,969	11.7	
**Follow-up years**^**a**^	7.42 (6.09–8.71)		7.40 (6.07–8.70)		0.094
**Newly diagnosed AD**	398	3.1	501	2.0	< 0.001*
**Newly diagnosed PD**	211	1.7	249	1.0	< 0.001*

^a^Indicates median (interquartile range); AD indicates Alzheimer disease; PD indicates Parkinson disease.

*Indicates statistical significance.

### Tinnitus on risks of Alzheimer disease

**Figure 1:**
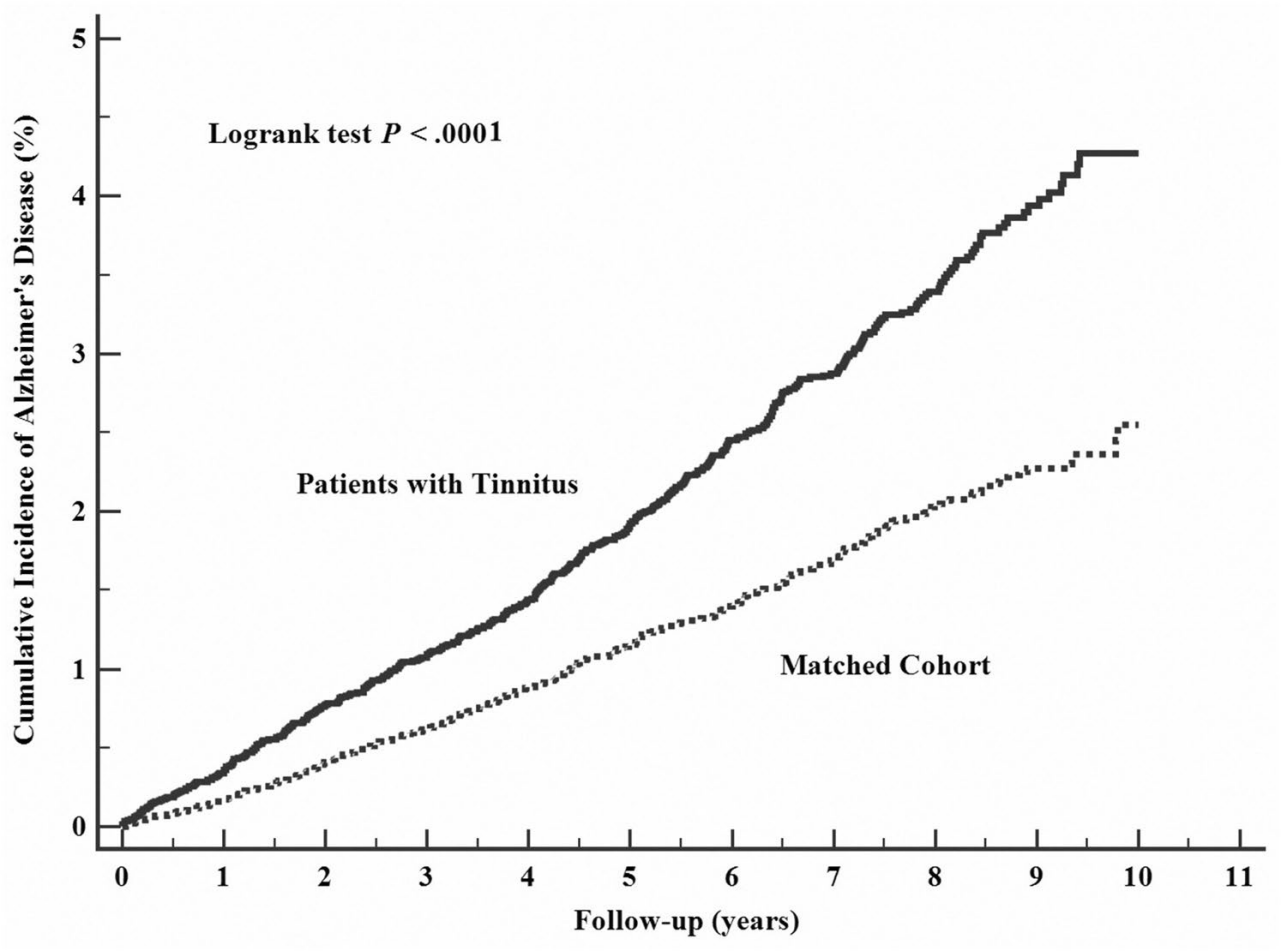
Cumulative incidence of Alzheimer's disease estimated for tinnitus group (solid line) and control group (dashed line).

The hazard ratio (HR) for developing AD in the follow-up period was higher for tinnitus patients when compared with control patients in three models (Model I: HR 1.62; Model II: HR 1.54; Model III: 1.34) (Table 2, Fig. 1). Furthermore, independent risk factors for AD included diabetes mellitus (HR 1.25, 95% CI 1.03–1.52, *P* = 0.027), and head injury (HR 1.66, 95% CI 1.35–2.04, *P* < 0.001). On the other hand, these were independent protective factors for AD when compared to low income, medium income (HR 0.77, 95% CI 0.63–0.95, *P* = 0.016) and when compared to urban, suburban (HR 0.80, 95% CI 0.66–0.97, *P* = 0.024) (Supplementary Table S1).

**Table 2:** Associations between tinnitus with risk of Alzheimer’s disease and Parkinson’s disease.

	Alzheimer’s disease		Parkinson’s disease	
	HR (95% CI)	*P* value	HR (95% CI)	*P* value
Model I	1.62 (1.41–1.85)	< 0.001	1.69 (1.41–2.04)	< 0.001
Model II	1.54 (1.34–1.78)	< 0.001	1.56 (1.29–1.89)	< 0.001
Model III	1.34 (1.17–1.53)	< 0.001	1.44 (1.19–1.73)	< 0.001

Model I: univariate analysis.

Model II: included univariate analysis factors with a moderate statistically significant relationship (i.e., *P* <  0.1) into a multivariate Cox proportional-hazards regression model by using the forward selection method.

Model III: included all variates.

### Tinnitus on risks of Parkinson disease

**Figure 2:**
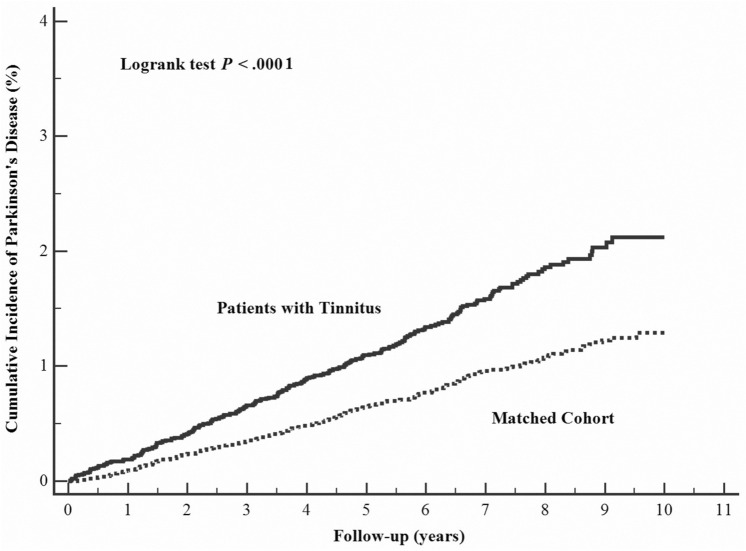
Cumulative incidence of Parkinson's disease estimated for tinnitus group (solid line) and control group (dashed line).

In comparison to control patients, the HR for developing PD during the follow-up period was higher for the tinnitus patients (Model I: HR 1.69; Model II: HR 1.56; Model III: 1.44) (Table 2, Fig. 2). Again, conditions such as head injury (HR 1.40, 95% CI, 1.06–1.85, *P* = 0.020), cerebrovascular disease (HR 1.52, 95% CI 1.08–2.14, *P* = 0.017), and osteoarthritis (HR 1.31, 95% CI 1.02–1.68, *P* = 0.033) were independent risk factors for PD (Supplementary Table S2).

### Tests of proportional-hazards assumption

The results of testing the proportionality of hazards in our work showed that there is no evidence that the proportional-hazards assumption has been violated in each cox regression models in our study (Supplementary Table S3).

## Discussion

To the best of our knowledge, this is the first nationwide, population-based retrospective cohort study evaluating the incidence of AD and PD in patients with tinnitus. The major findings in our study are that (1) the risks of developing AD and PD were increased after patients were newly diagnosed with tinnitus (HR 1.54 and 1.56); (2) diabetes mellitus, and head injury are independent risk factors to develop AD, and head injury, cerebrovascular disease, and osteoarthritis were independent risk factors for PD.

From the data provided by nuclear medicine imaging and fluorodeoxyglucose-positron emission tomography/computed tomography, the pathological neurodegenerative process in the central nervous system has been recognized in a specific cohort of patients (n = 16/96; 16.8%) diagnosed with a primary central type, severe, disabling subjective idiopathic tinnitus^6^. In addition, the possibility of neurodegenerative disease was indicated by the significance of subjective idiopathic tinnitus in a small particular cohort of patients (n = 16/18; 88.9%)^6^. There is also a high prevalence of cognitive difficulties reported among tinnitus patients from a clinical perspective^30^. As demonstrated by our study, tinnitus is an independent risk factor for subsequent PD and AD. Although the cause-effect relationship of tinnitus and AD/PD is unknown and even different, the occurrence of tinnitus may be a possible risk factor of AD/PD. Further research is required to validate our findings and investigate the pathophysiology about the relationship of tinnitus and Alzheimer’s disease and Parkinson’s disease. Moreover, whether prevention and treatment of tinnitus could be helpful in decreasing the incidence of AD/PD warrant further research. But, there are some possible mechanisms for exploring the association between tinnitus and AD/PD. Theoretically, neurodegeneration is contributed by inflammation, as it may be connected to and also precedes ischemia^6^. Tinnitus research finds that inflammation may be the trigger for the ringing in ears. The clinical features of tinnitus may be an extension of the primary pathological process of AD/PD^6^. Second, many etiological factors may be involved in the genesis of tinnitus, such as hyperactivity of the auditory cortex and dorsal cochlear nucleus, which may be damaged by oxidative stress. Besides, dysfunction between the central cortex and the inner ear is growingly considered to be linked to free radical–induced oxidative injury^31^. One of the primary causes of cell and tissue damage is lipid peroxidation generated by reactive oxygen species. The consequent lipid hydro-peroxide has been immunohistochemically marked in AD and PD in pathology^9^. As suggested by strong evidence, the pathogenesis of aging-related neurodegenerative diseases such as PD and AD, may be connected to raised oxidative stress^32^.

There is an independent association in the incidence of AD with diabetes mellitus, head injury in our study. Furthermore, an incidence of PD associated with head injury, cerebrovascular disease, and osteoarthritis were also found. Our findings were compatible with previous studies, indicating that diabetes mellitus^33^, and head injury^34^ were risk factors for developing AD and neurodegeneration. Compatible with previous reports, we also found that head injury^35^, cerebrovascular disease^36^, and osteoarthritis^37^ were related to higher risk of PD.

As the relationship between AD and the levels of monthly income was examined, it was found that medium monthly income may act as a relatively protective factor from AD development than those with low monthly income. In past studies^38–40^, higher education and socioeconomic status were associated with lower risk of Alzheimer’s disease. Another incidental finding was that suburban residency may be more protective from developing AD than urban residency. Although there were evidences that AD are associated with some environmental agents^41^, such as heavy metals or pesticides, the risk of developing AD related to the levels of urbanization was still not identified^42,43^.

Long follow-up duration, diagnosis of tinnitus by specialists, and large sample size are the strengths of our study. This current study design also included an unprejudiced participant selection process. Due to the ability of obtaining health care with low copayments, there is low referral bias and high follow-up compliance for all Taiwanese residents, and participation in the NHI is also mandatory.

There are some limitations to our findings. First, information regarding the family history of AD and PD, level of education, depression, physical activity, lifestyle factors (smoking, caffeine and alcohol consumption), and environmental factors (exposure to pesticides or heavy metals) were not acquired in the NHIRD, all of which are associated with the risk of neurodegenerative diseases^44^. Second, treatment for tinnitus may be associated with risk of Alzheimer’s disease and Parkinson’s disease, but these data were unknown in this study. Further research is warranted to determine whether these factors affect the risk of developing AD and PD. Third, the duration of the follow-up period in our study may have not been long enough to detect late-onset AD and PD. Thus, future studies with longer follow-up periods are required to clarify the long-term risk of AD and PD in patients with tinnitus.

## Conclusion

Our nationwide population-based retrospective cohort study implied that tinnitus patients had higher risk for developing AD and PD. Diabetes mellitus, and head injury increased the risk of developing AD. Also, head injury, cerebrovascular disease, and osteoarthritis raised the risk of subsequent PD. This information is critical for clinicians to develop preventive and diagnostic strategies for assessment.

## Supplementary information

Supplementary tables.

## Data Availability

The data that support the findings of this study are available from Taiwan National Health Insurance Research Database (NHIRD). To gain access, interested individuals should contact NHIRD.
