# Supplementary material for: Tinnitus and risk of Alzheimer’s and Parkinson’s disease: a retrospective nationwide population-based cohort study
Source: Sci Rep. 2020 Jul 22;10:12134. doi: 10.1038/s41598-020-69243-0 (PMC7376045; doi:10.1038/s41598-020-69243-0)
Supplement: Supplementary file 1 — Supplementary tables. [file 41598_2020_69243_MOESM1_ESM.docx]

**Tinnitus and risk of Alzheimer’s and Parkinson’s disease: A retrospective nationwide population-based cohort study**

Hsuan-Te Chu^1,2^, MD; Chih‐Sung Liang^1,4^, MD; Ta-Chuan Yeh^3^, MD; Li-Yu Hu^5,6^, MD; Albert C. Yang^2,6,7^, MD, PhD; Shih-Jen Tsai^2,5,6*^, MD; Cheng-Che Shen^6,8*^, MD, PhD

^1^ Department of Psychiatry, Beitou branch, Tri-Service General Hospital, National Defense Medical Center, Taipei, Taiwan

^2^ Institute of Brain Science, National Yang-Ming University, Taipei, Taiwan

^3^ Department of Psychiatry, Tri-Service General Hospital, National Defense Medical Center, Taipei, Taiwan

^4^ Graduate Institute of Medical Sciences, National Defense Medical Center, Taipei, Taiwan

^5^ Department of Psychiatry, Taipei Veterans General Hospital, Taipei, Taiwan

^6^ School of Medicine, National Yang-Ming University, Taipei, Taiwan

^7^ Division of Interdisciplinary Medicine and Biotechnology, Beth Israel Deaconess Medical Center/Harvard Medical School, Boston, Massachusetts, USA

^8^ Department of Psychiatry, Chiayi Branch, Taichung Veterans General Hospital, Chiayi, Taiwan

***Address correspondence to:** Dr. Cheng-Che Shen, M.D. (E-mail: [pures1000@yahoo.com.tw](mailto:pures1000@yahoo.com.tw)) or Dr. Shih-Jen Tsai, M.D. (E-mail: [tsai610913@gmail.com](mailto:tsai610913@gmail.com); Department of Psychiatry, Taipei Veterans General Hospital, Taipei, Taiwan (No 201, Sec 2. Shi-Pai Rd. Taipei, 11217, Taiwan.

Tel: +886-2-28757027 ext. 276; Fax: +886-2-28757592)

E-mail: tsai610913@gmail.com

**Supplementary table 1.** Analyses of Risk Factors for Alzheimer Disease in Patients with and without Tinnitus

| Predictive variables | | Univariate analysis | | | Multivariate analysis | | |
| --- | --- | --- | --- | --- | --- | --- | --- |
|  |  | HR (95% CI) | *P* value | | HR (95% CI) | *P* value | |
| Tinnitus | | 1.62 (1.41-1.85) | <.001 | 1.54 (1.34-1.78) | | <.001 |  |
| Comorbidities | |  |  |  | |  |  |
|  | Hypertension | 1.29 (1.09-1.53) | .004 | 1.06 (0.88-1.28) | | .544 |  |
|  | Diabetes mellitus | 1.41(1.17-1.69) | <.001 | 1.25 (1.03-1.52) | | .027 |  |
|  | Coronary artery disease | 0.88 (0.54-1.43) | .599 | |  |  | |
|  | Congestive heart failure | 1.27 (0.94-1.73) | .119 | |  |  | |
|  | Chronic lung disease | 1.40 (1.16-1.68) | <.001 | | 1.21 (0.99-1.47) | .059 | |
|  | Malignant neoplasms | 0.97 (0.62-1.53) | .909 | |  |  | |
|  | Head injury | 1.74 (1.43-2.12) | <.001 | | 1.66 (1.35-2.04) | <.001 | |
|  | Cerebrovascular disease | 1.21 (0.95-1.53) | .124 | |  |  | |
|  | Osteoarthritis | 1.32 (1.11-1.57) | .002 | | 1.11 (0.93-1.34) | .257 | |
|  | Rheumatologic disease | 1.45 (1.10-1.92) | .009 | | 1.19(0.89-1.60) | .246 | |
| Degree of urbanization | |  |  | |  |  | |
|  | Urban | Reference |  | | Reference |  | |
|  | Suburban | 0.76 (0.63-0.90) | .002 | | 0.80 (0.66-0.97) | .024 | |
|  | Rural | 0.70 (0.54-0.91) | .008 | | 0.80 (0.60-1.07) | .127 | |
| Income group | |  |  | |  |  | |
|  | Low income | Reference |  | | Reference |  | |
|  | Medium income | 0.72 (0.60-0.86) | <.001 | | 0.77 (0.63-0.95) | .016 | |
|  | High income | 0.80 (0.40-1.62) | .539 | | 0.95 (0.46-1.95) | .882 | |

HR indicates hazard ratio; CI indicates confidence interval;

**Supplementary table 2.** Analyses of Risk Factors for Parkinson Disease in Patients with and without Tinnitus

| Predictive variables | | Univariate analysis | | | Multivariate analysis | | |
| --- | --- | --- | --- | --- | --- | --- | --- |
|  |  | HR (95% CI) | *P* value | | HR (95% CI) | *P* value | |
| Tinnitus | | 1.69 (1.41-2.04) | <.001 | 1.56 (1.29-1.89) | | <.001 |  |
| Comorbidities | |  |  |  | |  |  |
|  | Hypertension | 1.44 (1.13-1.83) | <.001 | 1.16 (0.89-1.51) | | .279 |  |
|  | Diabetes mellitus | 1.34 (1.07-1.81) | .013 | 1.24 (0.94-1.63) | | .129 |  |
|  | Coronary artery disease | 1.00 (0.48-2.08) | .999 | |  |  | |
|  | Congestive heart failure | 0.94 (0.60-1.48) | .790 | |  |  | |
|  | Chronic lung disease | 1.41 (1.08-1.82) | .010 | | 1.20 (0.92-1.58) | .182 | |
|  | Malignant neoplasms | 0.91 (0.46-1.78) | .778 | |  |  | |
|  | Head injury | 1.51 (1.15-1.98) | .003 | | 1.40 (1.06-1.85) | .020 | |
|  | Cerebrovascular disease | 1.81 (1.31-2.51) | <.001 | | 1.52 (1.08-2.14) | .017 | |
|  | Osteoarthritis | 1.52 (1.20-1.92) | .001 | | 1.31 (1.02-1.68) | .033 | |
|  | Rheumatologic disease | 1.45 (1.00-2.10) | .049 | | 1.15 (0.78-1.70) | .492 | |
| Degree of urbanization | |  |  | |  |  | |
|  | Urban | Reference |  | |  |  | |
|  | Suburban | 1.02 (0.79-1.31) | .879 | |  |  | |
|  | Rural | 1.03 (0.74-1.45) | .859 | |  |  | |
| Income group | |  |  | |  |  | |
|  | Low income | Reference |  | |  |  | |
|  | Medium income | 0.99 (0.76-1.28) | .913 | |  |  | |
|  | High income | 0.85 (0.38-1.88) | .688 | |  |  | |

HR indicates hazard ratio; CI indicates confidence interval;

**Supplementary table 3.** Tests of proportional-hazards assumption

|  | Results for the global test |
| --- | --- |
| Parkinson’s disease | 0.2546 |
| Alzheimer’s disease | 0.1002 |
